# Physical Activity and Sedentary Behavior in Relation to Cancer Survival: A Narrative Review

**DOI:** 10.3390/cancers14071720

**Published:** 2022-03-28

**Authors:** Carmen Jochem, Michael Leitzmann

**Affiliations:** Department of Epidemiology and Preventive Medicine, University of Regensburg, Franz-Josef-Strauss-Allee 11, 93053 Regensburg, Germany; michael.leitzmann@ukr.de

**Keywords:** physical activity, sedentary behavior, cancer survival, review, recommendations, mortality

## Abstract

**Simple Summary:**

Globally, cancer is a major issue and an increasing number of people live with cancer. Lifestyle-associated factors play a major role in cancer prevention. Being physically active and limiting the amount of time spent sitting reduce the risk of developing several types of cancer. Furthermore, physical activity before, during, and after cancer diagnosis has been found to improve cancer outcomes. In addition, reduced levels of time spent sedentary may lead to improved outcomes in cancer survivors as well. This narrative review summarizes the existing evidence on the relationship of physical activity and sedentary behavior to cancer survival and other health outcomes in cancer survivors. The review provides an overview on the barriers, facilitators, and other factors that determine the levels of physical activity and sedentary behavior in cancer survivors as well as on the current recommendations on physical activity and sedentary behavior for cancer survivors.

**Abstract:**

From a public health perspective, cancer is a major issue, and it contributes to a high economic and societal burden. Lifestyle-associated risk factors play a crucial role in cancer prevention. The present narrative review aims to summarize the existing evidence on the relationship of physical activity and sedentary behavior to cancer survival, including the evidence on mortality and other health-related outcomes. There is strong evidence that physical activity before, during, and after cancer diagnosis improves outcomes for breast and colorectal cancers. In addition, there is emerging evidence that reduced levels of sedentary behavior in cancer survivors are associated with improved outcomes. Future studies are needed to strengthen the evidence and to provide details on additional cancer sites. In the meantime, existing recommendations for physical activity and sedentary behavior in cancer survivors should be followed to improve the health status of cancer survivors.

## 1. Introduction

Worldwide, the global burden of cancer is immense, and cancer is the second leading cause of death. Over 19 million new cancer cases, and approximately 10 million cancer deaths, occurred in 2020 (which included nonmelanoma skin cancers) [1]. The number of cancer cases is expected to increase by almost 50% by 2040 [1]. Demographic change (i.e., a growing and aging population), as well as highly prevalent risk factors, such as tobacco and alcohol consumption, physical inactivity and sedentary behavior, and an unhealthy weight and diet, contribute to the increasing cancer burden [2]. From a public health perspective, cancer is a major issue, and it contributes to a high economic and societal burden. Furthermore, improvements in cancer therapy, as well as detection at an early stage, lead to an increased number of cancer survivors (i.e., an increased number of people living with cancer). Thus, it is crucial to improve, promote, and maintain the physical and mental conditions of these cancer survivors.

**Figure 1:**
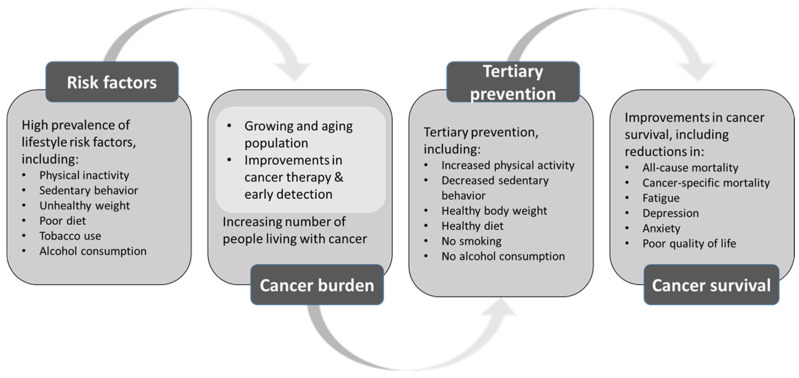
The role of lifestyle risk factors for the cancer burden, and the potential of tertiary prevention for improved cancer-related outcomes.

There is strong evidence that lifestyle-associated factors play a major role in the primary prevention of cancer. Being physically active and limiting the amount of time spent sitting reduce the risk of developing several types of cancer [3]. Although research on the roles of physical activity and sedentary behavior in cancer survival is less extensive, physical activity before, during, and after cancer diagnosis has been found to improve cancer outcomes [4]. In addition, emerging evidence shows that reduced levels of sedentary behavior lead to improved cancer outcomes [5]. Figure 1 shows the role of the lifestyle risk factors for the cancer burden, and the potential of tertiary prevention for improved cancer-related outcomes.

The current review aims to summarize: (i) The existing evidence on the relationship of pre- and postdiagnosis physical activity and sedentary behavior to cancer survival and other health outcomes in cancer survivors; (ii) The barriers, facilitators, and other factors that determine the levels of physical activity and sedentary behavior in cancer survivors; and (iii) The current recommendations on physical activity and sedentary behavior for cancer survivors.

## 2. Methods

By using information from meta-analyses, systematic reviews, and individual studies, we perform a narrative review that summarizes the current evidence on physical activity and sedentary behavior in relation to cancer recurrence and mortality, as well as the common and well-investigated side effects of cancer and cancer treatment, such as quality of life, fatigue, depression, pain, and the anthropometric outcomes in cancer survivors. Specifically, we searched PubMed for meta-analyses, systematic reviews, and individual studies that were published up to January 2022 by using the search terms: “physical activity”, “sedentary behavior”, “cancer survivor”, and “outcome.” In the following, we provide definitions for physical activity and sedentary behavior, and we describe the prevalence of those behaviors among cancer survivors. We report on the barriers, facilitators, and other factors that determine the levels of physical activity and sedentary behavior in cancer survivors. We also evaluate the quality of the evidence and outline future research needs.

## 3. Epidemiologic Evidence on Physical Activity and Cancer Survival

### 3.1. Definition of Physical Activity and Prevalence among Cancer Survivors

Physical activity is defined as any bodily movement that is produced by the skeletal muscles that requires energy expenditure [6]. There are different types of physical activities, as well as different degrees of activity intensity, frequency, and duration. Studies suggest that many cancer survivors are insufficiently physically active. Data from the Centers for Disease Control and Prevention show that 34% of cancer survivors aged 18 years or older reported no physical activity in their leisure time [7]. A study that objectively assessed physical activity in 1447 cancer survivors by using ActiGraph accelerometers revealed that, on average, the participants spent merely 3% of their time in moderate-to-vigorous physical activity, whereas they spent 66% of their time being sedentary. This finding suggests that considerable potential exists for cancer survivors to increase their moderate-to-vigorous activity and to decrease their sedentary behavior [8]. Older participants, women, and overweight or obese participants had significantly lower moderate-to-vigorous physical activity than their younger male normal-weight counterparts.

### 3.2. Physical Activity and Mortality Outcomes in Cancer Survivors

Several systematic reviews and meta-analyses have investigated the relationship between physical activity and the mortality outcomes in cancer survivors [4,9,10,11,12,13,14,15,16]. In the following, we report the main findings of the most recent and comprehensive systematic review and meta-analysis. Subsequently, we compare these findings to those of other studies, and we briefly discuss the similarities and differences between the studies.

A large systematic review and meta-analysis pooled the risk estimates of 136 studies that investigated the mortality outcomes among cancer survivors in relation to their levels of physical activity before and after cancer diagnosis [4]. The authors investigated all-cause mortality, cancer-specific mortality, and mortality due to cardiovascular disease (CVD). For the all-cause mortality, the higher compared to the lower levels of prediagnosis physical activity showed statistically significant reduced risks for the following cancer sites: colorectal cancer (HR = 0.80; 95% CI = 0.74−0.87); breast cancer (HR = 0.82; 95% CI = 0.76−0.87); hematologic cancer (HR = 0.84; 95% CI = 0.79−0.89); and prostate cancer (HR = 0.89; 95% CI = 0.82−0.98). The association between prediagnosis physical activity and all-cause mortality was not statistically significant in survivors of esophageal, female reproductive, and stomach cancers, nor in patients with melanoma [4]. For higher compared to lower levels of postdiagnosis physical activity, a statistically significant reduction in all-cause mortality was observed in cancer survivors with breast, colorectal, female reproductive, hematologic, kidney, lung, prostate, and stomach cancers, as well as in patients with glioma. The hazard ratios ranged between 0.58 (95% CI = 0.52−0.65) for breast cancer, and 0.76 (95% CI = 0.60−0.97) for lung cancer. A borderline statistical significance was observed in patients with childhood cancers (HR = 0.79; 95% CI = 0.62−1.00). The association between postdiagnosis physical activity and all-cause mortality in patients with esophagus cancer was not statistically significant [4].

For the cancer-specific mortality, the highest versus lowest levels of prediagnosis physical activity were associated with statistically significant risk reductions following stomach, liver, colorectal, lung, hematologic, and breast cancers. The hazard ratios ranged between 0.74 (95% CI = 0.58−0.95) for stomach cancer, and 0.86 (95% CI = 0.78–0.94) for breast cancer. Higher compared to lower postdiagnosis physical activity was protective against cancer-specific mortality following colorectal (HR = 0.62; 95% CI = 0.44−0.86), breast (HR = 0.63; 95% CI = 0.50−0.78), and prostate cancer (HR = 0.70; 95% CI = 0.55−0.90) diagnoses, at a statistically significant level [4].

For both the all-cause and cancer-specific mortality, the reductions in the magnitudes of the hazard ratios were more pronounced for the postdiagnosis than for the prediagnosis physical activity. Additional subgroup analyses by domain of physical activity showed consistent reductions in the magnitudes of the mortality hazards for both the total and recreational physical activity. The findings for other activity domains, including transportation, occupation, and household activity, were less studied and were inconsistent [4].

The studies of breast cancer that included dose–response analyses of physical activity showed inverse relationships between both the pre- and postdiagnosis physical activity and the breast-cancer-specific mortality and all-cause mortality, with steep reductions in the magnitudes of the mortality hazards for physical activity doses of 10–15 or more metabolic equivalent (MET) hours per week [4]. The MET is the resting metabolic rate that is achieved during quiet sitting [6]. A total of 10–15 MET hours per week correspond approximately to the levels of physical activity that are recommended by the World Health Organization (WHO). The reductions in the magnitudes of the mortality hazards were largest for postdiagnosis physical activity and all-cancer mortality, with recreational physical activity of 5, 10, 20, 30, and 65 metabolic equivalent hours per week, which led to reductions in the all-cause mortality by 22, 43, 59, 69, and 108%, respectively [4].

In addition to the all-cause and cancer-specific mortality, the association between the pre- and postdiagnosis physical activity (as a combined risk estimate) and the CVD mortality was investigated. The pooled risk estimates show a statistically significant reduction in the magnitudes of the mortality hazards for all-cancer (HR = 0.60; 95% CI = 0.50−0.73) and colorectal cancer (HR = 0.60; 95% CI = 0.40−0.91). The association was not statistically significant for childhood cancers (HR = 0.89; 95% CI = 0.49−1.61) [4].

The findings of this systematic review and meta-analysis [4] extend the results from previous systematic reviews and meta-analyses that focused primarily on physical activity in relation to survival after a breast [9,10,11,12,13] or colorectal cancer diagnosis [11,14]. These previous systematic reviews and meta-analyses include a smaller number of studies, but their pooled risk estimates were comparable in magnitude with the study by Friedenreich and colleagues [4]. For example, a meta-analysis that investigated the relationship between physical activity and mortality in breast cancer survivors included 16 cohort studies, and showed that high versus low levels of prediagnosis physical activity were associated with a relative risk (RR) of 0.81 (95% CI = 0.72−0.90) for breast-cancer-specific mortality, and of 0.76 (95% CI = 0.69−0.83) for all-cause mortality [9]. High versus low levels of postdiagnosis physical activity were associated with RRs of 0.68 (95% CI = 0.57−0.82) for breast-cancer-specific mortality, and of 0.52 (95% CI = 0.43−0.64) for all-cause mortality [9]. Although that meta-analysis included a smaller number of studies, the pooled risk estimates were similar to those of Friedenreich et al. [4], which included a larger number of studies.

Another meta-analysis that investigated the association between the physical activity and the colorectal cancer mortality in colorectal cancer survivors from 11 cohort studies showed that both pre- and postdiagnosis physical activity was associated with decreased overall and colorectal-cancer-specific mortality [14]. High versus low levels of prediagnosis physical activity were associated with a relative risk (RR) of 0.81 (95% CI = 0.72−0.91) for the overall mortality, and of 0.79 (95% CI = 0.71−0.89) for the colorectal-cancer-specific mortality. For the postdiagnosis physical activity, the pooled RRs were 0.71 (95% CI = 0.63−0.81) and 0.77 (95% CI = 0.63−0.94) for the overall mortality and the colorectal-cancer-specific mortality, respectively [14]. Compared to the findings of Friedenreich et al. [4], the pooled risk estimates are comparable but not identical, which may be explained by the larger number of studies that are included by Friedenreich et al.

In addition to the mortality outcomes in cancer survivors, the relation between physical activity and cancer recurrence or progression was investigated by several studies. The findings suggest that physical activity protects against recurrence and progression in breast cancer survivors. However, the evidence is not entirely clear because of the potential heterogeneity in the outcome measures [4].

### 3.3. Physical Activity and Other Health Outcomes in Cancer Survivors

Several systematic reviews summarize the relationship between physical activity and patient-reported outcomes, such as quality of life, fatigue, or depression.

Patient-reported outcomes, such as quality of life, play an important role for cancer survivors, and may be improved through physical activity. A meta-synthesis of 40 qualitative studies showed that physical activity in cancer survivors improved four dimensions of quality of life: physical, psychological, social, and spiritual well-being [17]. Fatigue is another common cancer-related side effect. A review summarized the findings of several systematic reviews and meta-analyses that investigated the relationship between physical activity and fatigue in cancer survivors, and it concluded that physical activity leads to modest improvements in fatigue [18]. With regard to the dose of physical activity, light and moderate intensities of physical activity seem to achieve the strongest improvements. Furthermore, physical activity has been shown to reduce depressive symptoms in cancer survivors [19].

### 3.4. Barriers, Facilitators, and Other Factors That Determine Levels of Physical Activity in Cancer Survivors

A systematic scoping review identified 98 articles that investigated the factors that influence the physical activity participation among cancer survivors [20]. In general, the cancer survivors reported positive attitudes towards physical activity participation, and they recognized the benefits of physical activity for physical and mental well-being. Among the physical activity preferences of individuals diagnosed with cancer, walking was the most preferred type of physical activity. Swimming, cycling, and yoga were also commonly reported types of physical activity. The majority of cancer survivors reported a preference for performing physical activity at home, without company, and in the morning. With regard to the source of information on physical activity, cancer survivors preferred oncologists, followed by physiotherapists, and nurses. The most preferred time to start physical activity programs was after treatment. However, varying preferences with regard to the activity type, place, and time, as well as preferences for individual versus group activity, point to the need for individualized physical activity programs for cancer survivors.

The barriers and facilitators to engagement in physical activity can be grouped into the following three thematic areas: physiologic factors; psychosocial and cultural factors; and economic and environmental factors [20]. These thematic areas also reflect the components of the Behavior Change Wheel and its accompanying framework of theoretical domains [21]. The main physiologic barriers to physical activity participation are cancer-related or treatment-related side effects (e.g., fatigue) and prevalent comorbidities [20]. In contrast, feeling well and engaging in effective symptom management facilitates physical activity participation. Psychosocial barriers include low self-efficacy and motivation. By comparison, perceived health benefits, positive previous experiences with physical activity, and social support or guidance by healthcare providers facilitate physical activity participation. With regard to the economic and environmental factors, financial unaffordability, inaccessible fitness facilities, and poor weather are commonly stated barriers. In contrast, the availability of affordable physical activity programs that are tailored to cancer survivors appears to be a strong facilitator of physical activity participation [20].

Several other systematic reviews and meta-analyses have investigated the barriers, facilitators, and correlates of physical activity in cancer survivors. Many of these studies focus on specific cancer sites (e.g., breast cancer survivors [22,23,24]), or specific stages of cancer treatment, but they reveal comparable factors, as is shown by the comprehensive systematic review by Elsahat et al. (2021) [20]. However, it is crucial to consider the factors that are specific to cancer sites and treatments in order to improve the physical activity participation of cancer survivors according to their particular situation.

In order to overcome the barriers in the physical activity engagement of cancer survivors, including the physiologic, psychosocial, cultural, economic, and environmental barriers, efforts need to be undertaken at different levels (e.g., from recommendations at the policy level, to implementations at the institutional level) and in different sectors (including not only the healthcare sector, but also the infrastructure/transport sector). For example, many physiologic factors may be improved through improved cancer symptom management. Psychosocial and cultural barriers may be overcome through innovative and integrative medical healthcare concepts that include physical activity and the corresponding counselling, guidance, and support during all stages of cancer treatment. With regard to the economic and environmental barriers, accessible, affordable, and inclusive community-based fitness facilities are needed in order to overcome the barriers in the physical activity participation among cancer survivors.

### 3.5. Quality of Evidence and Future Research Needs

There is strong evidence for the beneficial effects of physical activity before or after cancer diagnosis on cancer survival for breast and colorectal cancer [4]. The evidence is also clear for improved prostate-cancer-specific survival [4]. Furthermore, there is clear evidence that postdiagnosis physical activity is beneficial for cancer survival, independent of the prediagnosis physical activity levels [4]. However, the evidence with regard to the domain (i.e., recreation, transportation, occupation, and household activity) and the dose of physical activity in cancer survivors remains unclear [4].

In order to strengthen the current evidence, large randomized controlled exercise interventions with high-quality objective repeated assessments of the physical activity are necessary, that specifically address cancer sites other than breast, colorectal, and prostate cancers, and that target subsets of cancer survivors with distinct activity profiles. Research should also focus on the frequencies, doses, and domains of physical activity that are most beneficial for cancer survivors.

## 4. Epidemiologic Evidence on Sedentary Behavior and Cancer Survival

### 4.1. Definition of Sedentary Behavior and Prevalence among Cancer Survivors

Sedentary behavior is defined as, “any waking behavior characterized by an energy expenditure ≤1.5 metabolic equivalents, while in a sitting, reclining or lying posture” [25]. It is a highly prevalent behavior in people both with and without a diagnosis of cancer. A study that objectively assessed the sedentary behavior in 1447 cancer survivors by using ActiGraph accelerometers revealed daily sitting times of 9.5 h [8]. That study identified an older age, the male sex, and obesity as correlates of the time spent sedentary among cancer survivors. Furthermore, the time spent in sedentary bouts (i.e., sedentary bouts of ≥20 min without interruption) was, on average, 3.9 h per day. Older participants, males, overweight and obese cancer survivors, participants receiving a combined treatment of surgery, radiotherapy, and chemotherapy, as well as cancer survivors with higher-than-average fatigue, showed significantly greater time in sedentary bouts.

### 4.2. Sedentary Behavior and Mortality Outcomes in Cancer Survivors

A recent study evaluated the relation between sedentary behavior and the total and cancer-specific mortality by linking data from 2371 cancer survivors from the U.S. National Health and Nutrition Examination Survey (NHANES) with the U.S. mortality registry [26]. Spending 10 h per day or more in sedentary behaviors increased the risk of all-cause mortality in cancer survivors by 62% (HR = 1.62; 95% CI = 1.01–2.59). Furthermore, a monotonically increasing linear relationship between the sitting time and all-cause mortality showed that an increase of one standard deviation (= 187 min/day) of sitting time led to a 15% risk increase in all-cause mortality (HR = 1.15; 95% CI = 1.03–1.28). However, there was no significant association between the sitting time and cancer-specific mortality (HR = 1.10; 95% CI = 0.91–1.33).

In addition to this study, another study analyzed data from NHANES and jointly investigated the association between sedentary behavior and leisure-time physical activity with survival among U.S. cancer survivors Cao [27]. That study showed that spending more than eight hours per day in sedentary behaviors was associated with an increased risk of all-cause (HR = 1.81; 95% CI = 1.05–3.14) and cancer-specific (HR = 2.27; 95% CI = 1.08–4.79) mortality in cancer survivors, compared to lower amounts of sedentary behavior (less than four hours per day). The highest overall (HR = 5.38; 95% CI = 2.99–9.67) and cancer-specific (HR = 4.71; 95% CI = 1.60–13.9) mortality risks were observed in cancer survivors who were both highly sedentary and physically inactive.

Furthermore, a recent systematic review and meta-analysis [28] pooled the data from nine prospective cohort studies and they show that the risk of all-cause mortality was 22% higher in cancer survivors with higher versus lower levels of sedentary time after diagnosis (hazard ratio = 1.22; 95% CI = 1.06–1.41). Furthermore, pooled data from three prospective cohort studies show that most sedentary colorectal cancer survivors had a statistically significantly higher risk of colorectal-cancer-specific mortality compared to the least sedentary group (HR = 1.53; 95% CI = 1.14–2.06) [28]. A more detailed investigation of the same three prospective cohort studies examined the associations of sedentary behavior that was assessed pre- and postdiagnosis in relation to colorectal-cancer-specific mortality [5]. The association between sedentary behavior performed after colorectal cancer diagnosis and cancer-specific mortality was stronger (RR = 1.61; 95% CI = 1.23–2.11) than between sedentary behavior performed prior to colorectal cancer diagnosis (RR = 1.38; 95% CI = 1.08–1.75) [5].

Besides the abovementioned increased risk of colorectal-cancer-specific mortality in cancer survivors, individual studies that investigated cancer-specific mortality in survivors of renal [29], prostate [30], and hematologic [31] cancers did not show an association between the time spent sitting and cancer-specific mortality.

### 4.3. Sedentary Behavior and Other Health Outcomes in Cancer Survivors

In addition to the mortality outcomes in cancer survivors, the relationship between sedentary behavior and patient-reported outcomes, such as quality of life, fatigue, or depression, was summarized in a systematic review [28]. The findings from eleven studies on postdiagnosis sedentary behavior and quality of life showed that most studies (N = 7) reported null findings. However, four studies (one prospective and three cross-sectional studies) showed a statistically significantly lower quality of life among cancer survivors with higher levels of sedentary behavior. Studies that investigated the association between postdiagnosis sedentary behavior and fatigue in cancer survivors reported mixed results. Whereas one prospective and four cross-sectional studies showed a statistically significant positive association between sedentary behavior and fatigue, another prospective and five cross-sectional studies showed no association between the two. Furthermore, most of the studies that investigated the association between the postdiagnosis sitting time and anxiety or depression in cancer survivors showed null associations. In addition, the findings from two prospective and three cross-sectional studies show that postdiagnosis sedentary time appears to be unrelated to pain in cancer survivors.

Whereas the cross-sectional studies show no associations between postdiagnosis sedentary time and body mass index or waist circumference, the abovementioned systematic review [28] also summarized the findings from studies on the postdiagnosis sedentary time and anthropometric outcomes and identified one prospective study that showed a positive association between the television viewing time and the body mass index.

### 4.4. Interventions to Reduce Time Spent Sitting in Cancer Survivors

Several intervention studies aimed to reduce the time spent sedentary in cancer survivors. However, most of these interventions primarily targeted physical activity, and not sedentary behavior, and were performed in breast cancer survivors [32,33]. These studies did not show an effect on reducing sedentary behavior, which may be explained by the fact that they used exercise-based interventions, as well as by methodological limitations, such as the short-term durations of the interventions. Furthermore, sitting takes place in numerous contexts, such as television watching, working at a computer, driving a car, or socializing, and the sitting time in certain contexts may be more or less detrimental to cancer outcomes. Thus, it appears crucial to assess the specific circumstances during which the sitting among cancer survivors takes place in order to tailor specific interventions that address context-specific sitting.

### 4.5. Quality of Evidence and Future Research Needs

The quality of evidence with regard to the relations of postdiagnosis sedentary behavior and all-cause mortality and colorectal-cancer-specific mortality is reportedly low because the available data are limited to observational studies [28]. The quality of evidence for the patient-reported and anthropometric outcomes is low because of an inconsistency in the findings and the risk of bias. The quality of evidence for the effectiveness of interventions to reduce the sitting time in cancer survivors is not yet established, which may be due to the limited number of existing intervention studies in that particular study population.

In order to strengthen the evidence that is based on the sedentary behavior and all-cause mortality in cancer survivors, more intervention studies and well-designed prospective cohort studies are required, which minimize or avoid the potential of reverse causality (i.e., individuals with severe cancer histories potentially spending more time being sedentary). Furthermore, research should focus on the contexts in which the sedentary behavior takes place, as well as the interaction between the sedentary behavior and the physical activity in cancer survivors.

## 5. Existing Recommendations for Physical Activity and Sedentary Behavior for Cancer Survivors

The WHO Guidelines on Physical Activity and Sedentary Behavior strongly recommend that cancer survivors “limit the amount of time spent being sedentary”, and “replace sedentary time with physical activity of any intensity (including light intensity)” [34]. The WHO further recommends that they “do more than the recommended levels of moderate- to vigorous-intensity physical activity” in order to “reduce the detrimental effects of high levels of sedentary behaviour on health” [34]. However, the evidence that underlies these recommendations is of low certainty because population-specific studies that investigate the association between the sedentary behavior and health outcomes in people with chronic diseases, including cancer, are still scarce. Therefore, the WHO extrapolated the evidence from the general adult population to adults with chronic conditions.

The WHO physical activity recommendations for adults with chronic conditions, including cancer survivors, are more specific and are of moderate certainty. The WHO strongly recommends that cancer survivors “undertake regular physical activity” and “do at least 150–300 min of moderate-intensity aerobic physical activity; or at least 75–150 min of vigorous-intensity aerobic physical activity; or an equivalent combination of moderate and vigorous-intensity activity throughout the week”. Furthermore, cancer survivors should “also do muscle-strengthening activities at moderate or greater intensity that involve all major muscle groups on 2 or more days a week”. Older adults living with cancer “should do varied multicomponent physical activity that emphasizes functional balance and strength training at moderate or greater intensity on 3 or more days a week, to enhance functional capacity and prevent falls”. For additional health benefits, it is recommended that adult and older adult cancer survivors increase the duration, frequency, and intensity of physical activity—provided there are no contraindications.

Beyond the WHO guidelines, several other evidence-based recommendations exist and will be briefly summarized here. For cancer survivors, the World Cancer Research Fund/American Institute for Cancer Research (WCRF/AICR) recommend being “physically active as part of everyday life—walk more and sit less” [35]. The American Cancer Society (ASC) provides evidence-based general recommendations on physical activity for cancer survivors [36]: “Physical activity assessment and counseling should begin as soon as possible after diagnosis, with the goal of helping patients prepare for treatments, tolerate and respond to treatments, and manage some cancer-related symptoms and treatment-related side effects.” In order to improve long-term health, the ACS recommendation is to “avoid obesity and maintain or increase muscle mass through diet and physical activity”, and to “engage in regular physical activity, with consideration of type of cancer, patient health, treatment modalities, and symptoms and side effects”. The European Society for Clinical Nutrition and Metabolism [37] provides two exercise recommendations for all cancer survivors: “We recommend maintenance or an increased level of physical activity in cancer patients to support muscle mass, physical function and metabolic pattern. We suggest individualized resistance exercise in addition to aerobic exercise to maintain muscle strength and muscle mass.” [37]. Evidence-based recommendations with regard to physical activity (and sedentary behavior) are also provided by the American Society of Clinical Oncology (ASCO) [38,39,40,41], the National Comprehensive Cancer Network (NCCN) [42], and other national physical activity and sedentary behavior guidelines (e.g., Physical Activity Guidelines for Americans [43]).

## 6. Conclusions and Opinion

Both physical inactivity and sedentary behavior are highly prevalent behaviors in cancer survivors, despite increasing evidence that increased physical activity and decreased sedentary behavior are beneficial for the health outcomes in this population. In our opinion, regular exercise needs to become an integral component of cancer treatment. To that end, healthcare providers and exercise specialists need to more actively help to educate cancer survivors to “move more than less”. Specifically, patients need to be instructed to begin their exercise program slowly, with gradual increases, and to avoid any activity that poses a risk for falls or injury. Physical activities should include aerobic and strength components and may be complemented by stretching. Making physical activity a part of their daily routine, and keeping exercise easy and enjoyable, will help patients to engage in longer-term adherence to being more active and less sedentary. Examples include using the stairs instead of the elevator or escalator, walking the dog, and playing active games with kids. Such activities may also help relieve stress, anxiety, and depression. In addition, cancer survivors should be empowered to “sit less”. Therefore, strategies that target sedentary behavior reductions in cancer survivors should be developed and implemented.
